# Gastrodin Ameliorates Motor Learning Deficits Through Preserving Cerebellar Long-Term Depression Pathways in Diabetic Rats

**DOI:** 10.3389/fnins.2019.01239

**Published:** 2019-11-22

**Authors:** Cheng-Kun Deng, Zhi-Hao Mu, Yi-He Miao, Yi-Dan Liu, Lei Zhou, Yong-Jie Huang, Fan Zhang, Yao-Yi Wang, Zhi-Hong Yang, Zhong-Yi Qian, Xie Wang, Jia-Zhi Guo, Mei-Yan Zhang, Xin-Yu Liao, Qi Wan, Di Lu, Ying-Ying Zou

**Affiliations:** ^1^Department of Pathology and Pathophysiology, Faculty of Basic Medical Sciences, Kunming Medical University, Kunming, China; ^2^Department of Thoracic Surgery, First Affiliated Hospital of Kunming Medical University, Kunming, China; ^3^Department of Orthopedics, The Fifth Affiliated Hospital, Kunming Medical University, Kunming, China; ^4^Institute of Drug Discovery and Development, Kunming Pharmaceutical Corporation, Kunming, China; ^5^The Key Laboratory of Stem Cell and Regenerative Medicine of Yunnan Province, Kunming Medical University, Kunming, China; ^6^Emergency Department, First Affiliated Hospital of Kunming Medical University, Kunming, China; ^7^The Second Affiliated Hospital of Kunming Medical University, Kunming, China; ^8^Department of Morphological Laboratory, Faculty of Basic Medical Sciences, Kunming Medical University, Kunming, China; ^9^Biomedical Engineering Research Center, Kunming Medical University, Kunming, China; ^10^Institute of Neuroregeneration and Neurorehabilitation, Department of Neurosurgery of the Affiliated Hospital, Qingdao University, Qingdao, China

**Keywords:** diabetes, cerebellum, Purkinje cells, LTD, gastrodin

## Abstract

Cognitive dysfunction is a very severe consequence of diabetes, but the underlying causes are still unclear. Recently, the cerebellum was reported to play an important role in learning and memory. Since long-term depression (LTD) is a primary cellular mechanism for cerebellar motor learning, we aimed to explore the role of cerebellar LTD pathways in diabetic rats and the therapeutic effect of gastrodin. Diabetes was induced by a single injection of streptozotocin into adult Sprague–Dawley rats. Motor learning ability was assessed by a beam walk test. Pathological changes of the cerebellum were assessed by Hematoxylin-Eosin (HE) and Nissl staining. Cellular apoptosis was assessed by anti-caspase-3 immunostaining. Protein expression levels of LTD pathway-related factors, including GluR2, protein kinase C (PKC), NR2A, and nNOS, in the cerebellar cortex were evaluated by western blotting and double immunofluorescence. The NO concentration was measured. The cellular degeneration and the apoptosis of Purkinje cells were evident in the cerebellum of diabetic rats. Protein expression levels of GluR2 (NC9W: 1.26 ± 0.12; DM9W + S: 0.81 ± 0.07), PKC (NC9W: 1.66 ± 0.10; DM9W + S: 0.58 ± 0.19), NR2A (NC9W: 1.40 ± 0.05; DM9W + S: 0.63 ± 0.06), nNOS (NC9W: 1.26 ± 0.12; DM9W + S: 0.68 ± 0.04), and NO (NC9W: 135.61 ± 31.91; DM9W + S: 64.06 ± 24.01) in the cerebellum were significantly decreased in diabetic rats. Following gastrodin intervention, the outcome of motor learning ability was significantly improved (NC9W: 6.70 ± 3.31; DM9W + S: 20.47 ± 9.43; DM9W + G: 16.04 ± 7.10). In addition, degeneration and apoptosis were ameliorated, and this was coupled with the elevation of the protein expression of the abovementioned biomarkers. Arising from the above, we concluded that gastrodin may contribute to the improvement of motor learning by protecting the LTD pathways in Purkinje cells.

## Introduction

Diabetes mellitus (DM) is a multifactorial disease characterized by chronic metabolic disturbances. It has been reported to cause several pathological complications, such as retinopathy, nephropathy, and neuropathy (Lv et al., 2018). Increasing evidence has shown that diabetes can lead to cognitive dysfunction, including impairments in learning and memory (Guven et al., 2009), dysfunctions in execution (Kodl and Seaquist, 2008), and motor coordination (Cox et al., 2005). Furthermore, diabetic patients have an increased risk of vascular dementia (O’Brien and Thomas, 2015), Alzheimer’s disease (Dominguez et al., 2014), and Parkinson’s disease (De Pablo-Fernandez et al., 2018). However, the underlying mechanism of DM-induced cognitive dysfunction remains unclear.

The cerebellum is closely linked to the brainstem and spinal cord, and it has long been recognized as a center for motor coordination and control (Stoodley, 2012). However, the cerebellar role in motor functioning has overshadowed the development of insights in the causal relationship between cerebellar pathology and a variety of neurocognitive deficits (Noroozian, 2014). Several studies in humans and animals have shown that the cerebellum plays an important role in cognitive processing, motor learning, and memory (Gonzalez-Tapia et al., 2015; Lawrenson et al., 2018). Since long-term depression (LTD) at the parallel fibers associated with the Purkinje cell synapses within the cerebellar cortex has been considered as a primary cellular mechanism for cerebellar motor learning (Inoshita and Hirano, 2018), we aimed to investigate the effect of diabetes on the cerebellum and the role of LTD pathways in DM-induced cognitive dysfunction.

The most common neurotransmitter involved in LTD is L-glutamate, which acts on α-amino-3-hydroxy-5-methyl-4-isoxazolepropionic acid receptors (AMPARs) and N-methyl-D-aspartate receptors (NMDARs). Importantly, this intrinsic plasticity was based on intracellular Ca^2+^ signaling and protein kinase C (PKC) pathways (Piochon et al., 2010; Galliano et al., 2018). Therefore, we postulated that the abovementioned factors related to LTD may play an important role in cerebellar motor learning.

Gastrodin, a phenolic glycoside chemically known as 4-hydroxyphenyl-beta-d-glucopyranoside, is the main active component of *Gastrodia elata*. With a molecular weight of 286 Da, it can pass through the blood–brain barrier and be detected in the cerebellum. Interestingly, the concentration of gastrodin in the cerebellum was found to be higher than in other brain regions in the central nervous system (Wang et al., 2008). After entering into the central nervous system, gastrodin can improve learning and memory capacity in neurological diseases like Alzheimer’s disease (Hu et al., 2014), Parkinson’s disease (Tansey and Goldberg, 2010), and vascular dementia (Li and Zhang, 2015) by regulating neurotransmitters and reducing microglial activation as well as inflammation. However, the effect of gastrodin on cerebellar motor learning and LTD pathways has remained elusive.

In light of the above, we hypothesized that gastrodin could improve motor learning and memory deficits in DM through protecting Purkinje cells and preserving LTD pathways, which might be reflected by factors including PKC, NR2A, GluR2, and nNOS. This study sought to assess the effect of gastrodin on Purkinje cells and the relevant factors in streptozotocin-induced diabetes.

## Materials and Methods

### Induction of Diabetes

Seventy male SD rats (10-week-old, weight 250–300 g) were given the rodent diet and water *ad libitum.* Animal procedures were reviewed and approved by the Medical Ethics Committee of Kunming Medical University, Kunming, China. After 2 weeks of adaptation, type 1 diabetes was induced by a single intraperitoneal injection of 60 mg/kg of streptozotocin prepared in a 1% [w/v] solution of 0.1 M citrate buffer (pH 4.5) to the rats. Control rats received the same volume of sterile saline. Diabetes was assessed 72 h later by using a glucometer and animals were considered as diabetic if the blood glucose levels were higher than 16.7 mmol/L for three consecutive tests (Verhagen et al., 2018).

### Drug Administration

The rats were randomly divided into three groups (Lv et al., 2018). The NC9W group were normal control rats gavaged with normal saline daily (4 ml/kg) and fed for 6 weeks; (Guven et al., 2009) the DM9W + S group were diabetic rats which were gavaged with normal saline for 6 weeks at 3 weeks after diabetes induction; (Kodl and Seaquist, 2008) and the DM9W + G group were diabetic rats which were gavaged with gastrodin (60 mg/kg daily; dissolved in 0.9% saline) for 6 weeks (Qi et al., 2019).

### Beam Walk Test

Rats were trained to undergo motor coordination assessment by a narrow square wooden beam, 1 m long and 0.5 cm wide (Shaw et al., 2013). The beam was elevated 50 cm above the ground for the rats to return to their home cage. The rats were placed in the dark experimental room to acclimatize for 60 min and the temperature was kept constant. The rats were then placed at the start of beam and the latency to traverse the beam (up to 60 s) was recorded. Rats were trained for four sessions per day for four consecutive days. Finally, the latency time for the rats to cross the beam three times was assessed, and the values obtained were averaged.

### Western Blotting Analysis

The rats were anesthetized with 10% chloral hydrate administered intraperitoneally. The cerebellar tissues were rapidly dissected and immediately frozen in liquid nitrogen and stored in −80°C. Proteins were extracted from the cerebellar tissues by RIPA buffer (9806; Cell Signaling Technology) containing a 1% protease inhibitor cocktail (1:100; 5871; Cell Signaling Technology) and 1% phosphatase inhibitor cocktails (1:100; 5870; Cell Signaling Technology) at 4°C. Homogenates were centrifuged at 12,000 × *g* for 10 min, and the supernatant was collected. Protein concentration was measured using a BCA protein assay kit. The proteins (30 μg) were loaded unto SDS-PAGE gel. The gels were electrophoresed and then transferred to PVDF membranes. After that, the membranes were blocked with a blocking buffer using 5% non-fat milk for 120 min and probed with primary antibodies overnight at 4°C. They were then incubated for 2 h at room temperature with appropriate secondary mouse antibodies (1:1,000, Thermo Fisher Scientific). The following primary antibodies were used for this study: mouse anti-GluR2 antibody (1:1,000 dilution; Abcam), mouse anti-PKC antibody (1:1,000 dilution; Abcam), mouse anti-NR_2_A antibody (1:500 dilutions; Abcam), mouse anti-nNOS antibody (1:1,500 dilution; BD Biosciences), and β-tubulin (1:1,000, Cell Signaling Technology). The blots were developed with enhanced chemiluminescence and densitometric analysis of the film was accomplished with ImageJ software (version 1.4.3.67).

### Double Immunofluorescence

The cerebellum was dissected, immersed in 4% formaldehyde, dehydrated, cleared with xylene, and embedded in paraffin blocks. Paraffin sections of 4 μm thickness were deparaffinized and hydrated through a series of graded alcohol. The tissues were incubated in citrate buffer for antigen retrieval and the slices were incubated with 5% normal goat serum. The following primary antibodies were used: rabbit anti Calbindin D-28k antibody (1:1,000; Swant); mouse anti-GluR2 antibody (1:500, Abcam), mouse anti-PKC antibody (1:750; Abcam), mouse anti-nNOS antibody (1:200; BD Biosciences), and rabbit anti-active-caspase-3 antibody (1:100; Millipore). Primary antibodies were added in a fresh blocking solution and incubated overnight at 4°C. The staining was visualized with anti-mouse and anti-rabbit Alexa Fluor 488 and 568 2nd antibodies (1:500; Invitrogen, A11010, A11001). After washing in PBS, secondary antibodies were added in PBS containing 0.1% Triton X-100 to prevent non-specific antigen binding for 2 h at room temperature. Tissue sections were viewed, and images captured on an Olympus FV1000 microscope.

### Histological Study

To observe the histological changes, the sections were first incubated with hematoxylin for 5 min and then washed with 1% ethanol hydrochloride for 3 s. After rinsing with water, the sections were stained with eosin. After this, the sections were examined under a light microscope at the magnification ×400 in a blinded manner.

Nissl staining was performed by incubating the deparaffinized sections with Nissl staining solution containing cresyl violet (Beyotime Institute of Biotechnology, Shanghai, China) for 10 min at room temperature.

### Measurement of Nitric Oxide Production

The supernatant derived from the fresh cerebellar tissues was centrifugated. Total nitric oxide (NO) production was estimated by spectrophotometric measurement of nitrite and nitrate concentrations in the supernatant. For this, the procedure for Griess reagent with the Total Nitric Oxide Assay Kit (Beyotime Institute of Biotechnology, Jiangsu, China) was followed. An optical density at 540 nm was measured using a microplate reader. Concentrations were calculated by comparing absorptions with a standard curve.

### Statistical Analysis

Data were expressed as mean ± SD and analyzed with SPSS 17.0 statistical software (SPSS, United States). Comparisons among groups were performed using an analysis of variance, and pairwise comparison was performed using the LSD-*t* test. ^∗^*p* < 0.05 and ^∗∗^*p* < 0.01 was considered statistically significant.

## Results

### Gastrodin Improved Motor Learning in Diabetic Rats

**FIGURE 1:**
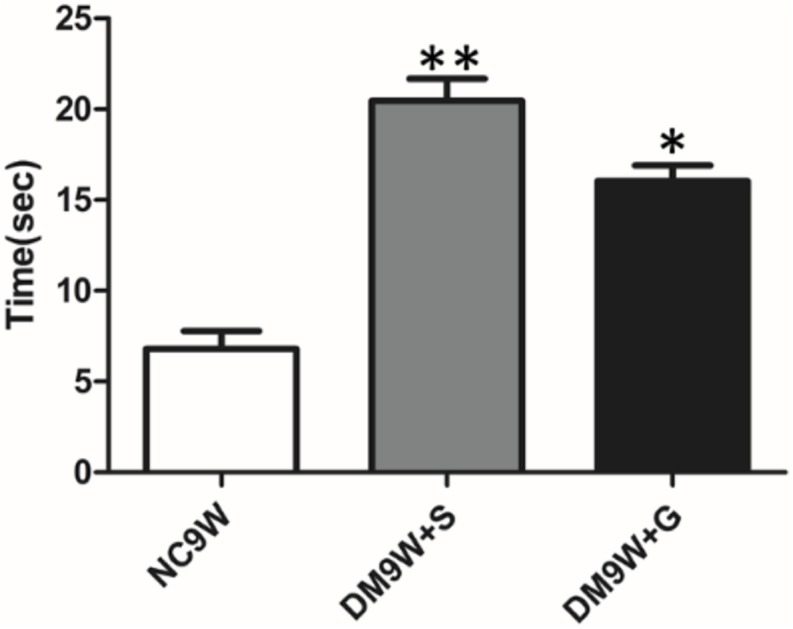
Alternation in beam-walking ability following STZ-induced diabetes. ^∗∗^*p* < 0.01 for comparison of diabetic group with the controls; ^∗^*p* < 0.05 for comparison of gastrodin intervention group with the controls. Note the significant improvement in latency time in the gastrodin treatment group (DM9W + G) when compared with the DM9W + S group.

Motor learning was assessed as total length of time spent by the animals on a narrowing beam (Figure 1). The latency time to cross the beam was significantly increased in the DM9W + S group, indicating the impairment of motor learning. Remarkably, gastrodin treatment significantly reduced the duration of time in diabetic rats (cf. DM9W + G and DM9W + S group).

### Gastrodin Ameliorated Pathological Changes of the Cerebellum and Protected Purkinje Cells in Diabetic Rats

**FIGURE 2:**
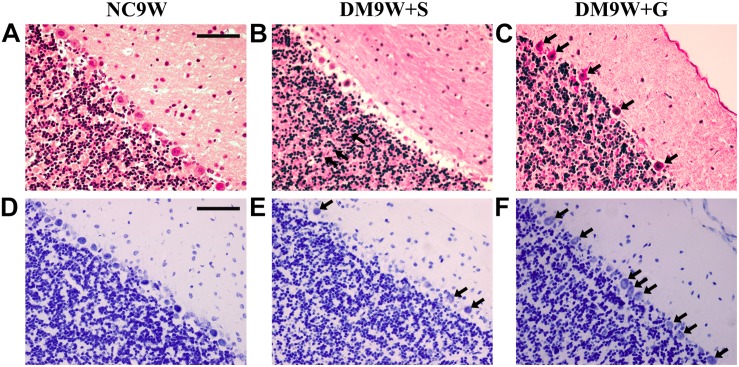
Effects of gastrodin on DM-induced pathological changes in the cerebellar cortex. H&E staining **(A–C)** and Nissl staining **(D–F)** of the cerebellar cortex in NC9W, DM9W + S, and DM9W + G groups. Black arrow indicates Purkinje cells. Note the drastic reduction of Purkinje neurons in the DM9W + S group. Note also the wide interstitial spaces around the Purkinje cells in the same group. In DM9W + G, the incidence of Purkinje cells is increased; moreover, the neuropil is now more compact. Magnification: ×400. Bar = 50 μm.

H&E staining showed that the Purkinje cells in the NC group were orderly arranged in a single row of cells. The cells were characterized by a round nucleus with discrete chromatin clumps (Figures 2A–C). However, in the DM9W + S group, the Purkinje cells were distributed in a haphazard manner and the cell number was markedly reduced. As a result, wide interstitial spaces indicative of edematous were observed in areas in what were supposed to be occupied by the Purkinje neurons as seen in the NC group. In the DM9W + G group, the external morphology and arrangement of Purkinje cells were comparable to those in the NC9W group, though fewer in number when compared with the NC group; moreover, the neutrophil around the Purkinje neurons appeared compact in the NC group.

The results with Nissl staining were consistent with that of H&E staining (Figures 2D–F). In the NC9W group, Nissl bodies were well defined in the Purkinje cells; however, in DM9W + S, Nissl bodies were hardly detected in the Purkinje cells. In the DM9W + G group, the number of Purkinje cells was increased; furthermore, the Nissl bodies contained in the Purkinje cells became more evident as in the NC9W group.

**FIGURE 3:**
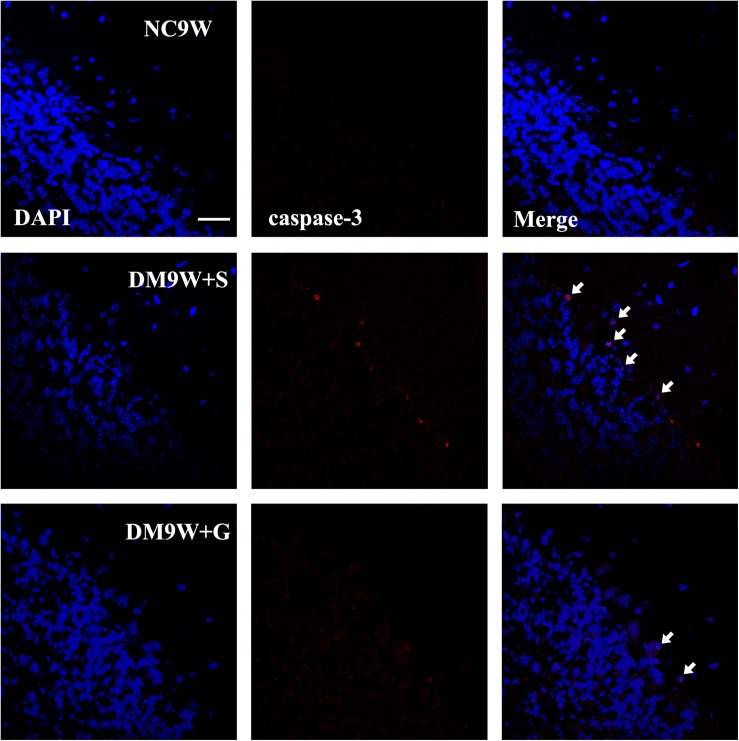
Representative photomicrographs of DAPI (blue) and caspase-3 (red) double staining of the cerebellum in the NC9W, DM9W + S, and DM9W + G groups. Note the increase in incidence of caspase-3 positive cells in DM9W + S (single arrow) compared with the NC9W group. However, in the DM9W + G group, caspase 3 + cells are hardly encountered. Magnification: ×600. Bar = 20 μm.

Immunostaining of caspase-3 revealed diabetes-induced cell death in the cerebellar cortex as evident by the identification of positive cells located primarily in the monolayer Purkinje cells. In the DM9W + G group, caspase 3 immunofluorescence was hardly detected (Figure 3).

### Gastrodin Enhanced Expression of Long-Term Depression-Related Markers in the Cerebellum of Diabetic Rats

**FIGURE 4:**
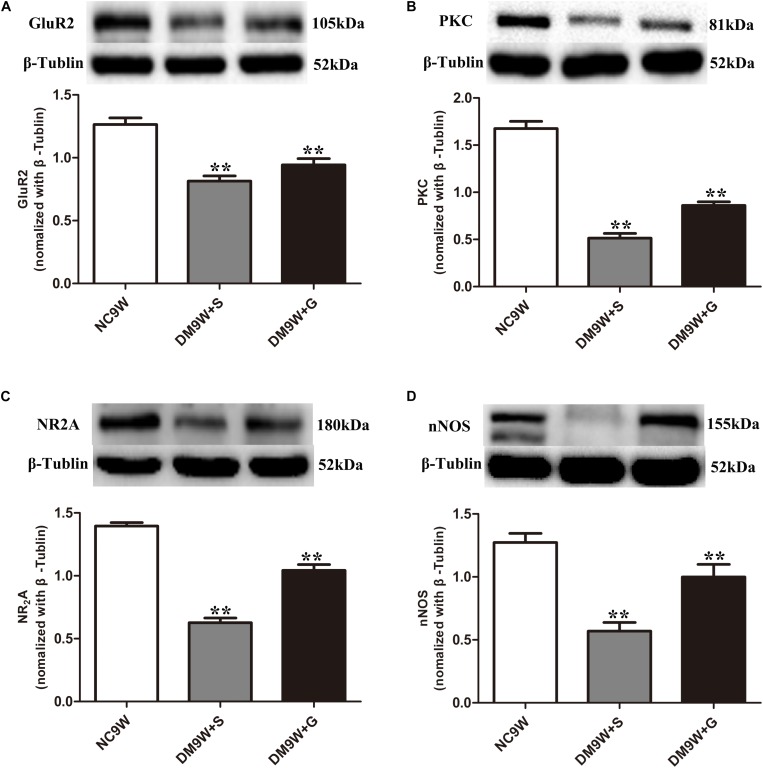
Western blot analysis of GluR2 **(A)**, PKC **(B)**, NR2A **(C)**, and nNOS **(D)** protein expression levels in the cerebellum in the NC9W, DM9W + S, and DM9W + G groups, including the immunoreactive bands of GluR2 (105 kDa), PKC (81 kDa), NR2A (180 kDa), nNOS (155 kDa), and β-Tubulin (52 kDa). Bar graphs representing optical density (mean ± SD). Note the expression levels of all biomarkers were significantly decreased after diabetes induction (DM9W + S) but were significantly reversed after gastrodin intervention (DM9W + G). ^∗∗^*p* < 0.01.

The protein expression of GluR2 was significantly decreased in the DM9W + S group, which was significantly reversed after gastrodin intervention (DM9W + G group) (*p* < 0.01). In parallel to this, protein expression levels of NR2A, PKC, and nNOS were significantly decreased in the DM9W + S group in comparison to the NC group (*p* < 0.01); the expression levels of these biomarkers in the DM9W + G group were significantly higher than in the DM9W + S group (Figure 4).

**FIGURE 5:**
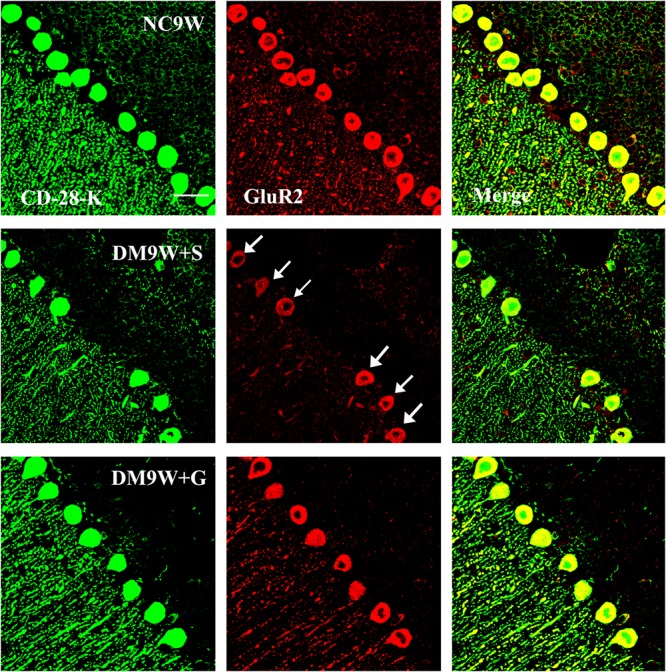
Representative photomicrographs showing CD-28K (green) and GluR2 (red) double immunofluorescence staining of the cerebellum in NC9W, DM9W + S, and DM9W + G groups. Note the drastic reduction of GluR2/CD28k + Purkinje cells in DM9W + S (single arrow) whose number was regained in DM9W + G. Magnification: ×600. Bar = 20 μm.

**FIGURE 6:**
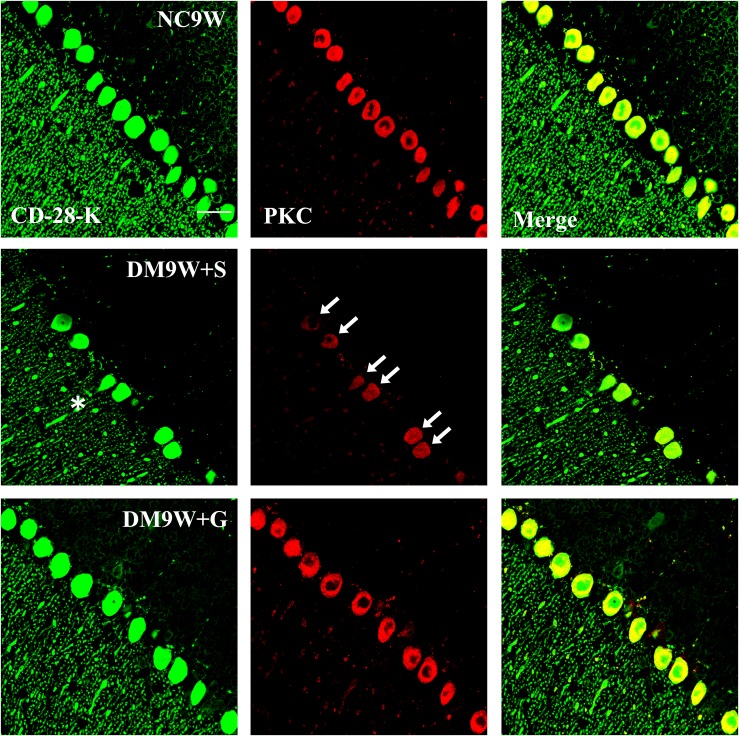
Representative photomicrographs showing CD-28K (green) and PKC (red) double immunofluorescence staining of the cerebellum in NC9W, DM9W + S, and DM9W + G groups. PKC-positive Purkinje cells double labeled with CD-28K were drastically reduced in DM9W + S (single arrow). This was accompanied by diminution of dendritic profiles in the cerebellar cortex (asterisk). In the DM9W + G group, the number of Purkinje cells showing colocalization of CD-28K and PKC was comparable to that in NC9W. Magnification: ×600. Bar = 20 μm.

**FIGURE 7:**
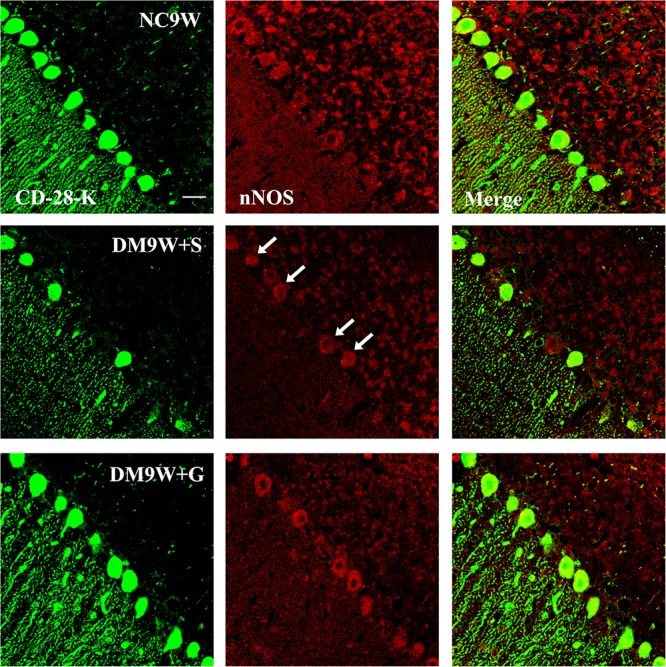
Representative photomicrographs showing CD-28K (green) and nNOS (red) double-labeled Purkinje cells in the cerebellum in the NC9W, DM9W + S, and DM9W + G groups. Note the diminution of nNOS immunofluorescence in Purkinje cells in the DM9W + S group (single arrow) as compared with the normal control (NC9W). nNOS immunofluorescence, however, was restored to a level comparable to that of the normal in DM9W + G. Magnification: ×600. Bar = 20 μm.

Double immunofluorescence labeling showed that GluR2 was localized primarily in the cell body and projecting apical dendrites of the cerebellar Purkinje cells. Note that in the NC9W and DM9W + G groups, the Purkinje cells emitted intense GluR2 immunofluorescence that was markedly attenuated in the DM9W + S group. Furthermore, the number of Purkinje cells was decreased in the DM9W + S group; this was coupled with decreased dendrites and their ramifications (Figure 5). Similar expression changes in PKC and nNOS immunofluorescence were observed in the Purkinje cells in the above groups (Figures 6, 7). Of note, besides the Purkinje cells, such expression was also detected in other cells of the cerebellar cortex.

### Gastrodin Increased NO Concentration

**FIGURE 8:**
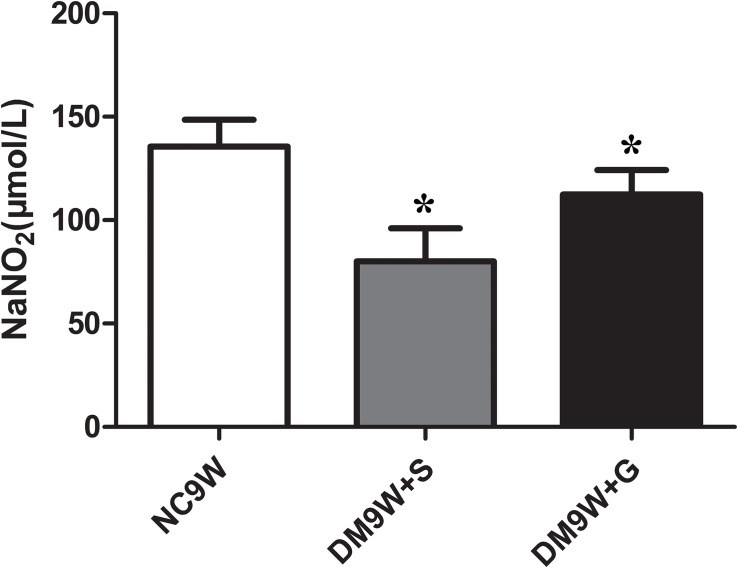
Determination of extracellular release of NO in NC9W, DM9W + S, and DM9W + G groups. Note the drastic decline in NO level in DM9W + S. Bar graphs represent NO concentration (mean ± standard deviation). ^∗^*p* < 0.05.

Compared with the NC9W + S group, NO level in the cerebellar tissue was significantly decreased in the DM9W + S group. In DM rats given gastrodin treatment, the level of NO rose sharply to a level that was not only higher than that in DM9W + S group but also exceeded by five folds that of the NC9W group (Figure 8).

## Discussion

This present study has shown that experimentally induced diabetes in adult rats could result in cerebellar damage and impairments in motor learning. More importantly, we have shown that gastrodin could protect Purkinje cells and preserve LTD pathways, which may account for the improvement of motor learning. Although many studies have reported diabetes-induced cerebellar damage and motor function deficiency (Hernandez-Fonseca et al., 2009; Nagayach et al., 2014; OzdemIr et al., 2016), the role of cerebellar LTD pathways in diabetes-induced cognitive dysfunction remains elusive. We have provided morphological and biochemical evidence to suggest that NMDAR-dependent and AMPAR-dependent LTD in the cerebellum was compromised due to Purkinje cell injury. This supports the therapeutic effects of gastrodin on motor learning and cerebellar LTD pathways.

LTD occurs in many areas of the CNS with varying mechanisms, and it has been best characterized in the cerebellum and hippocampus (Massey and Bashir, 2007). As a unique type of synaptic plasticity, cerebellar LTD was exhibited by Purkinje cells and regulated by various neurotransmitters, especially NMDARs and AMPARs, and was reported to be critical for motor learning. Separately, it has been reported that optogenetic control of synaptic AMPA receptor endocytosis directly inhibited cerebellar motor learning during adaptation of the horizontal optokinetic response and vestibulo-ocular reflex, supporting the direct effect of cerebellar LTD on motor learning (Kakegawa et al., 2018). Additionally, it has been shown that chronic neuroinflammation in LPS-infused rats could reduce postsynaptic NMDAR-dependent and AMPAR-dependent LTD and thus lead to memory impairment (Min et al., 2009). Furthermore, modulation of NMDAR function with an NMDA open channel blocker, memantine, was reported to rescue hippocampal LTD and improve spatial learning in juvenile-onset diabetic rats (Sacai et al., 2014). Furthermore, it has been shown that inhibition of hippocampal LTD, not LTP, impaired spatial memory consolidation (Ge et al., 2010). However, there are many studies on the relation between hippocampal LTD impairment and cognitive dysfunction in diabetes (CID), though the role of cerebellar LTD as well as its cellular basis has remained elusive. We postulated that restoration of cerebellar LTD pathways in diabetes would be a promising therapeutic target in CID.

In the present study, we found that diabetes induction caused increased latency to cross a beam, which indicated that the diabetic rats had motor-learning deficits. Remarkably, with gastrodin intervention, the latency was decreased in diabetic rats, indicating that gastrodin may be effective in treating CID. To assess the changes of LTD pathways, we examined the expression changes of its related biomarkers comprising NR2A, GluR2, PKC, nNOS, and NO in normal and experimental rats. We found that the protein expression level of NR2A was decreased in the cerebellum of diabetic rats. As a subunit of NMDAR, a reduced level of NR2A is reported to be associated with cognitive deficits as well as affective symptoms of depression (Feyissa et al., 2009). NR2A-containing NMDARs may contribute to the cerebellar LTD. As a subunit of AMPAR, GluR2 also plays a critical role in the induction of LTD (Isaac et al., 2007; Zhou et al., 2011). PKC is found to contribute to cerebellar LTD induction by phosphorylating GluR2 (Chung et al., 2003). In addition, evidence has suggested that NMDAR-mediated Ca^2+^ influx activates nNOS and ultimately regulates LTD by NO (Shin and Linden, 2005). The present results have shown that all the abovementioned factors were decreased in diabetic rats and were increased after gastrodin treatment. This suggests that impairment in cerebellar LTD pathways may be caused by the downregulation of both AMPAR and NMDAR and that gastrodin can improve the motor learning through increasing their protein expression level, thus preserving the cerebellar LTD pathways.

Purkinje cells are the primary relay neurons of the cerebellum and play an important role in motor learning and coordination. Cerebellar motor learning relies on movement errors signaled by climbing-fiber inputs to cause LTD of synapses from parallel fibers to Purkinje cells (Yang and Lisberger, 2014). Optogenetic activation of Purkinje cells can contribute to the induction of motor learning, assessed by a vestibulo-ocular reflex test (Nguyen-Vu et al., 2013). In diabetic rats, previous studies have found that prominent loss of cerebellar Purkinje cells may be attributed to inflammation and oxidative stress (Solmaz et al., 2017; Verhagen et al., 2018), which may be mediated by glia cells, e.g., microglial activation (Hu et al., 2014). In the present study, we have detected prominent damage of Purkinje cells in diabetic rats, including the loss of cell numbers, diminution of Nissl bodies, dendritic loss etc. A major finding in this study was the demonstration that gastrodin could protect the Purkinje cells and increase their cell number through inhibiting apoptosis, as was evident by the reduced immunostaining of caspase-3. Double immunostaining of GluR2, PKC, and nNOS with CD28K (a specific marker for Purkinje cells) showed that diabetes reduced the expression of these factors specifically in the Purkinje cells. A decrease in the above markers induced by diabetes, however, was suppressed by gastrodin. In light of the above, we suggest that gastrodin can exert its therapeutic effects on CID by reducing apoptosis of Purkinje cells as well as preserving the cerebellar LTD pathways, though the underlying protective mechanism of gastrodin on Purkinje cells remains to be explored.

Gastrodin has been extensively investigated in recent years in view of its anti-inflammatory and antioxidant properties that may be beneficial in the treatment of neurodegenerative diseases. Indeed, it has been extensively explored as a potential candidate drug for the treatment of CNS disorders because of its low toxicity (Zhan et al., 2016) and ability to pass through the BBB. It can be detected in the brain 5 min after *i.v.* administration (50 mg/kg). Although the brain-to-blood distribution ratio of gastrodin is relatively low, it was metabolized into *p*-Hydroxybenzyl alcohol, which has similar pharmacological effects (Liu et al., 2018).

Gastrodin has also been widely used as an analgesic, anti-inflammatory, antioxidative, and sedative agent (Zhao et al., 2012; Zhang et al., 2016). Moreover, a few clinical trials have shown that gastrodin could improve the neurological assessment of patients with cognitive dysfunction, such as in vascular dementia (Liu et al., 2018). However, the application of gastrodin on CID is dismal in view of the lack of a fuller understanding of its underlying biochemical mechanism. Besides the CNS system, gastrodin was reported to have effects on the circulatory system and the digestive system. It could effectively inhibit the formation of clots, exerting an anticoagulant function (Zhan et al., 2016). A recent study has found that gastrodin has lipid-regulating effects and thus could be used to treat non-alcoholic fatty liver disease (Ahmad et al., 2019).

The present study has shown that gastrodin can ameliorate diabetes-related Purkinje cell apoptosis and restore cerebellar LTD pathways; however, some limitations remain. First, electrophysiological analysis should have been conducted to confirm the changes of cerebellar LTD. Secondly, further investigations, such one into the effects of gastrodin on synaptic plasticity, would be desirable. Additionally, it would be of interest to examine the effect of gastrodin on astrocytes, which are closely associated with the regulation of glutamate levels in the microenvironment.

## Conclusion

**FIGURE 9:**
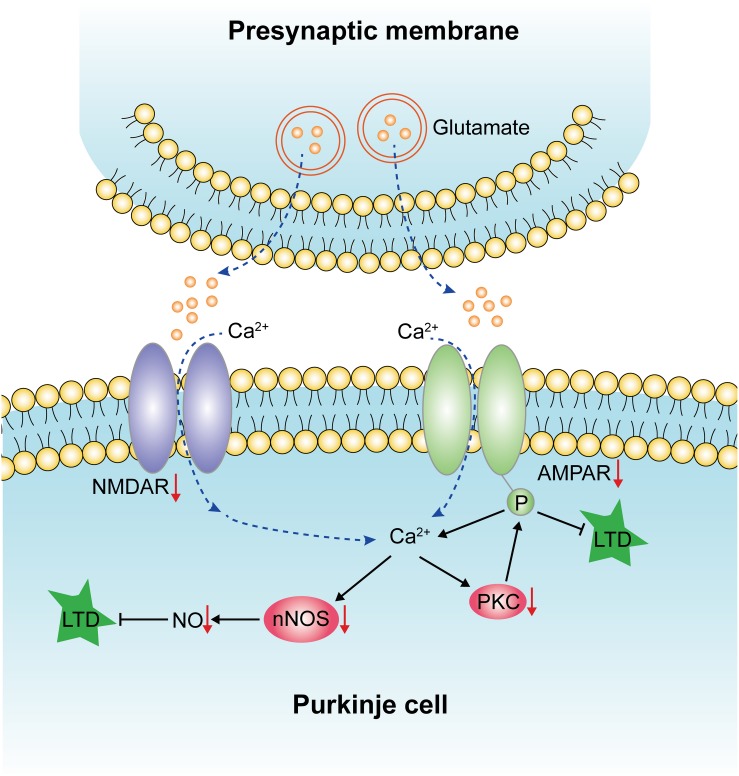
Diagram illustrating the effects of diabetes on LTD, which has been indicated by red arrows. Diabetes could reduce the expression of NMDAR and AMPAR, which affects the influx of calcium and results in decreased protein expression levels of nNOS, NO, and PKC. The downregulation of these factors leads to the impairment of LTD and, finally, the deficits of motor learning.

The present results have shown unequivocally that STZ-induced diabetes resulted in the increased apoptosis of Purkinje cells in the cerebellum. It is suggested that the death of Purkinje cells in diabetic rats may be related to the decreased expression of NMDAR, AMPAR, nNOS, and NO. It is speculated that this may affect the LTD (Figure 9). This mechanism may account for motor learning deficits of diabetic rats. More importantly, we have shown that gastrodin can ameliorate the incidence of apoptotic Purkinje cells and improve the motor learning ability of the diabetic rats, most probably through modulating the LTD pathways, namely NMDA receptor-dependent and AMPA receptor-dependent pathways.

## Data Availability Statement

The raw data supporting the conclusions of this manuscript will be made available by the authors, without undue reservation, to any qualified researcher.

## Ethics Statement

Animal procedures were reviewed and approved by the Medical Ethics Committee of Kunming Medical University, Kunming, China.

## Author Contributions

Y-YZ and DL designed the project, contributed to the analysis of data, and finalization of the manuscript. C-KD, Z-HM, and Y-HM performed the majority of the experiments, participated in the discussion and analysis of data, and prepared the first draft of the manuscript. Y-DL designed and guided the use of gastrodin. LZ, Y-JH, FZ, and Y-YW conducted parts of the experiments, and participated in discussion and analysis of the data. Z-HY, Z-YQ, and XW performed the paraffin embedding, sectioning, and H&E staining. J-ZG, M-YZ, and X-YL helped with the removal of tissue samples and took care of the experimental rats. QW revised the manuscript. Y-YZ is the corresponding author and DL is the co-corresponding author of this manuscript.

## Conflict of Interest

Y-DL was employed by Kunming Pharmaceutical Corporation. The remaining authors declare that the research was conducted in the absence of any commercial or financial relationships that could be construed as a potential conflict of interest.
